# Calls of *Boanalatistriata* (Caramaschi & Cruz, 2004) (Amphibia, Anura, Hylidae), an endemic tree frog from the State of Minas Gerais, Brazil

**DOI:** 10.3897/zookeys.820.30711

**Published:** 2019-01-29

**Authors:** Cyro de Luna-Dias, Sergio P. de Carvalho-e-Silva

**Affiliations:** 1 Laboratório de Anfíbios e Répteis. Departamento de Zoologia, Instituto de Biologia, Universidade Federal do Rio de Janeiro (UFRJ). Av. Universidade Federal do Rio de Janeiro Rio de Janeiro Brazil; 2 Carlos Chagas Filho, 373 – Prédio do CCS, Bloco A, Sala A1-111, Cidade Universitária, CEP 21941-902, Rio de Janeiro, RJ, Brazil Universidade Federal do Rio de Janeiro Rio de Janeiro Brazil

**Keywords:** Cophomantini, *Boanapulchella* species group, communication, bioacoustics, taxonomy

## Abstract

Bioacoustical data are useful for studying amphibians, especially their conservation, taxonomy, and evolution, among others. Of the 12 species of the *Boanapolytaenia* clade, only *B.buriti* and *B.latistriata* have no published information about their advertisement calls. We recorded four males of *B.latistriata* in its type locality at Parque Nacional do Itatiaia, south-eastern Brazil. We used a Roland R26 digital recorder with a Sennheiser ME-67 microphone and analysed the recordings using the Raven Pro 1.5 software. We recorded two different types of calls (call A and call B). Both were composed of one pulsed note and presented a slightly ascending-descending frequency modulation. Call A was more frequent, having durations between 0.042 and 0.093 s with the dominant frequency ranging from 3375.0 to 3937.5 Hz, and was composed of 11 to 21 pulses separated by intervals that were not fully silent. Call B had durations between 0.711 and 1.610 s, with dominant frequency from 3281.2 to 3750.0 Hz, and was composed of 11 to 29 pulses separated by fully silent intervals. Among the *B.polytaenia* clade, the calls of *B.latistriata* are more similar to those of *B.bandeirantes*, *B.beckeri*, *B.polytaenia*, and B.aff.beckeri. The calls of *B.latistriata* differ from these species in its lower dominant frequency. *Boanalatistriata* present a short, single-note call with a lower pulse period (call A) and a long call with higher pulse period (call B), which differ from the other species of the clade. The coefficients of variation for the various bioacoustical attributes were calculated within- and between-males and these have been discussed. We also report a fight event between two males of *B.latistriata*. This is the first report of a fight in members of the *B.polytaenia* clade.

## Introduction

Vocalization plays an essential role during the reproductive period of anurans, being species-specific and constituting a pre-zygotic mechanism of reproductive isolation (Duellman and Trueb 1994, Wells 2007). This makes bioacoustical data useful for studying topics such as conservation (Laiolo 2010, Forti et al. 2017), taxonomy (Hepp and Carvalho-e-Silva 2011, Carvalho and Giaretta 2013, Rivadeneira et al. 2018), social interaction (Giasson and Haddad 2006), and evolution (Robillard et al. 2006, Goicoechea et al. 2010). Amongst the different anuran call types, advertisement calls have the highest value in taxonomy (Köhler et al. 2017). Despite being the most studied call type, there is a need for further documentation (Toledo et al. 2014, Guerra et al. 2018a) and understanding of the importance of advertisement call variation both within- and between-individuals (Gerhardt 1991, Köhler et al. 2017).

The *Boanapolytaenia* clade is composed of 12 species of tree frogs with a striped dorsal pattern (Caramaschi and Cruz 2013): *B.bandeirantes* (Caramaschi & Cruz, 2013); *B.beckeri* (Caramaschi & Cruz, 2004); *B.botumirim* (Caramaschi, Cruz & Nascimento, 2009); *B.buriti* (Caramaschi & Cruz, 1999); *B.cipoensis* (Lutz, 1968); *B.goiana* (Lutz, 1968); *B.jaguariaivensis* (Caramaschi, Cruz & Segalla, 2010); *B.latistriata* (Caramaschi & Cruz, 2004); *B.leptolineata* (Braun & Braun, 1977); *B.phaeopleura* (Caramaschi & Cruz, 2000); *B.polytaenia* (Cope, 1870); and *B.stenocephala* (Caramaschi & Cruz, 1999). Of these, there is a lack of information on the advertisement calls of *B.buriti* and *B.latistriata* alone, while the information for *B.cipoensis* and *B.leptolineata* is very limited (Kwet 2001, Batista et al. 2015).

*Boanalatistriata* was described from Brejo da Lapa, an artificial pond in the Parque Nacional do Itatiaia (PNI), Itamonte Municipality, State of Minas Gerais, Brazil (Caramaschi and Cruz 2004). The type series of this species also includes five individuals from Marmelópolis, in the same state, which is the only record of the species outside the PNI. It is the largest species of the *B.polytaenia* clade, with the males measuring 34.9–40.6 mm and females 40.9–51.6 mm (Caramaschi and Cruz 2004). Tadpoles of this species have been described by Orrico et al. (2007). Toledo and Haddad (2009) have reported the distress call of one female of this species. The lack of information about *B.latistriata* led IUCN to list it as being “Data Deficient” (Stuart 2018), thus supporting the need to study this species. In this paper, we describe the advertisement call of *B.latistriata* from its type locality and report an event of a combat between males of this species.

## Material and methods

Recordings were made at Brejo da Lapa (22.3589°S, 44.7372°W, 2140 m altitude) on 12 November 2014, from 20:30 to 23:00. Vocalizations from four males were recorded with a Roland R26 digital recorder and a Sennheiser ME-67 shotgun microphone positioned between 20 and 50 cm from the calling males. After the calls were recorded, the specimens were collected, anesthetised and euthanized with 5% lidocaine, fixed in 10% formaldehyde, and subsequently preserved in 70% ethanol. These have been deposited in the amphibian collection of the Departamento de Zoologia, Instituto de Biologia, Universidade Federal do Rio de Janeiro (ZUFRJ), with collection numbers 15073, 15074, 15076, and 15077. Recordings were deposited at Fonoteca Neotropical Jacques Vielliard (FNJV; https://www2.ib.unicamp.br/fnjv/) with the respective numbers 40238, 40239, 40240 and 40241. All procedures were conducted under licence No. 40371 issued by the Instituto Chico Mendes de Conservação da Biodiversidade – ICMBio.

Recordings were made using 24 bits of resolution and 48 kHz sampling rate. Sound analyses were performed using the Raven Pro 1.5 software (Bioacoustics Research Program 2014) using window type = Hann, size = 256 samples, and overlap = 99%. Measurements and terminology follow Köhler et al. (2017). Terminology used to describe calls of the species belonging to the *B.polytaenia* clade (calls, notes and pulses) is highly variable (for example, see Acioli and Toledo 2008, Pinheiro et al. 2012, Martins et al. 2016). To keep nomenclature stability, we opted to follow the most recent papers on this topic (e.g. Martins et al. 2016, Guerra et al. 2017) and adopted a call centred approach (Köhler et al. 2017).

The following parameters were measured or calculated: call duration (CD), pulse duration (PD), pulse period (PP), interval between “call A” and “call B” (ABI), call dominant frequency (DF), call fundamental frequency (FF; measured through the “Peak Frequency” function of Raven Pro), number of visible harmonics (NH; integer multiples of the fundamental frequency), first pulse dominant frequency (FPDF), central pulse dominant frequency (CPDF, measured in the central pulse with higher dominant frequency), last pulse dominant frequency (LPDF), pulse rise time (RT; measured through the “Max Time” function of Raven Pro), proportion of pulse rise time in relation to pulse duration (RTR), and pulse number (PN). Measurements are given as a range, followed by mean and standard deviation (SD). For DF, FPDF, CPDF, LPDF, and PN, the mode (Mo; the most frequent value among the measurements) is also presented.

To determine the variation in the bioacoustical attributes, we calculated the within-individual coefficient of variation (CV_w_; Gerhardt 1991) for each parameter of each male. Between-individual coefficient of variation (CV_b_) was calculated for each parameter by pooling the measurements from all the males. Parameters with a coefficient of variation below 5.0% were considered static, whereas those with a coefficient of variation above 12.0% were considered dynamic (Gerhardt 1991).

## Results

Males of *B.latistriata* called from dusk (about 18:00) to at least 02:00, perched on shrubs and grass, near or above the water. At the time of the recordings, we visually counted more than 20 males of *B.latistriata* and heard many more at a distance. During the fieldwork, the weather was rainy, air temperature between 12 and 14 °C, and humidity above 90%. Other amphibian species recorded at the site were *Aplastodiscusalbosignatus* (Lutz & Lutz, 1938), *Bokermannohylagouveai* (Peixoto & Cruz, 1992), *Rhinellaicterica* (Spix, 1824), and *Scinaxduartei* (Lutz, 1951).

**Figure 1.**
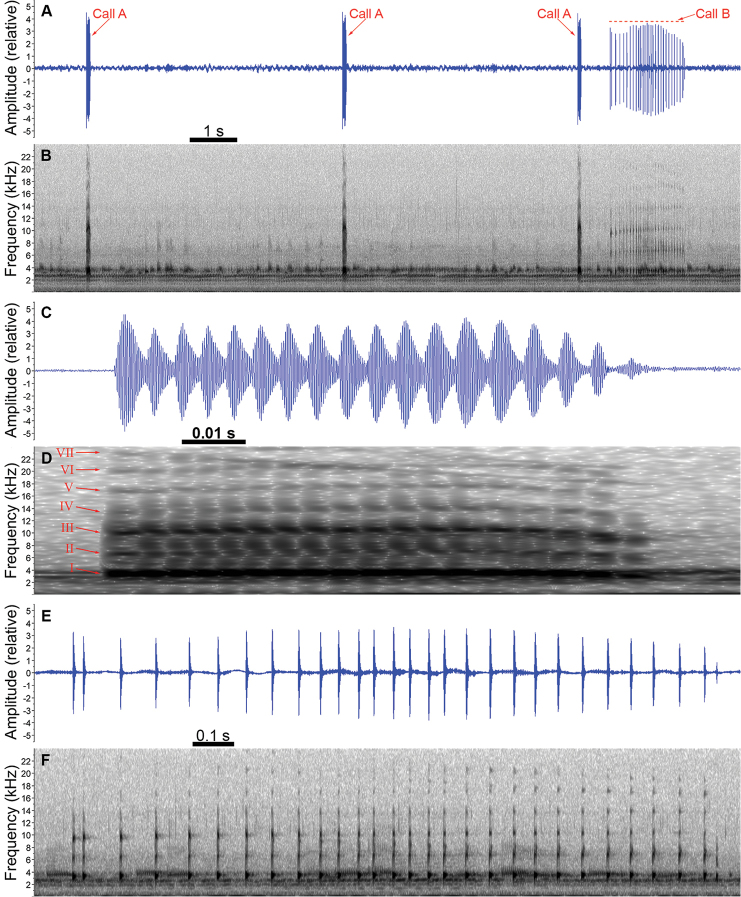
Calls of *Boanalatistriata* from its type locality. **A** Waveform and **B** spectrogram showing three instances of call A and one of call B emitted in sequence **C** waveform and **D** spectrogram of a call A, in detail, indicating seven visible harmonics numbered I to VII **E** waveform and **F** spectrogram of a call B, in detail. Images were obtained using Raven Pro 1.5 software. Spectrograms parameters: window type = Hann, size = 256 samples, overlap = 99%. Individual ZUFRJ 15076 (snout-vent length = 39.3 mm), recorded at a temperature between 12 and 14 °C.

We recorded two types of calls from the males of *B.latistriata*, herein called “call A” and “call B” (Fig. 1). Call A was emitted more frequently (103 of 142 recorded calls in the complete dataset), and we interpreted it as an advertisement call due to the social context in which it was emitted (frequently emitted even by isolated males, without aggressive interactions). Call B was always emitted after call A, with ABI varying from 0.545 to 1.622 s (mean = 0.782 s, SD = 0.231, n = 38 intervals), and possibly had some degree of territorial function.

**Figure 2.**
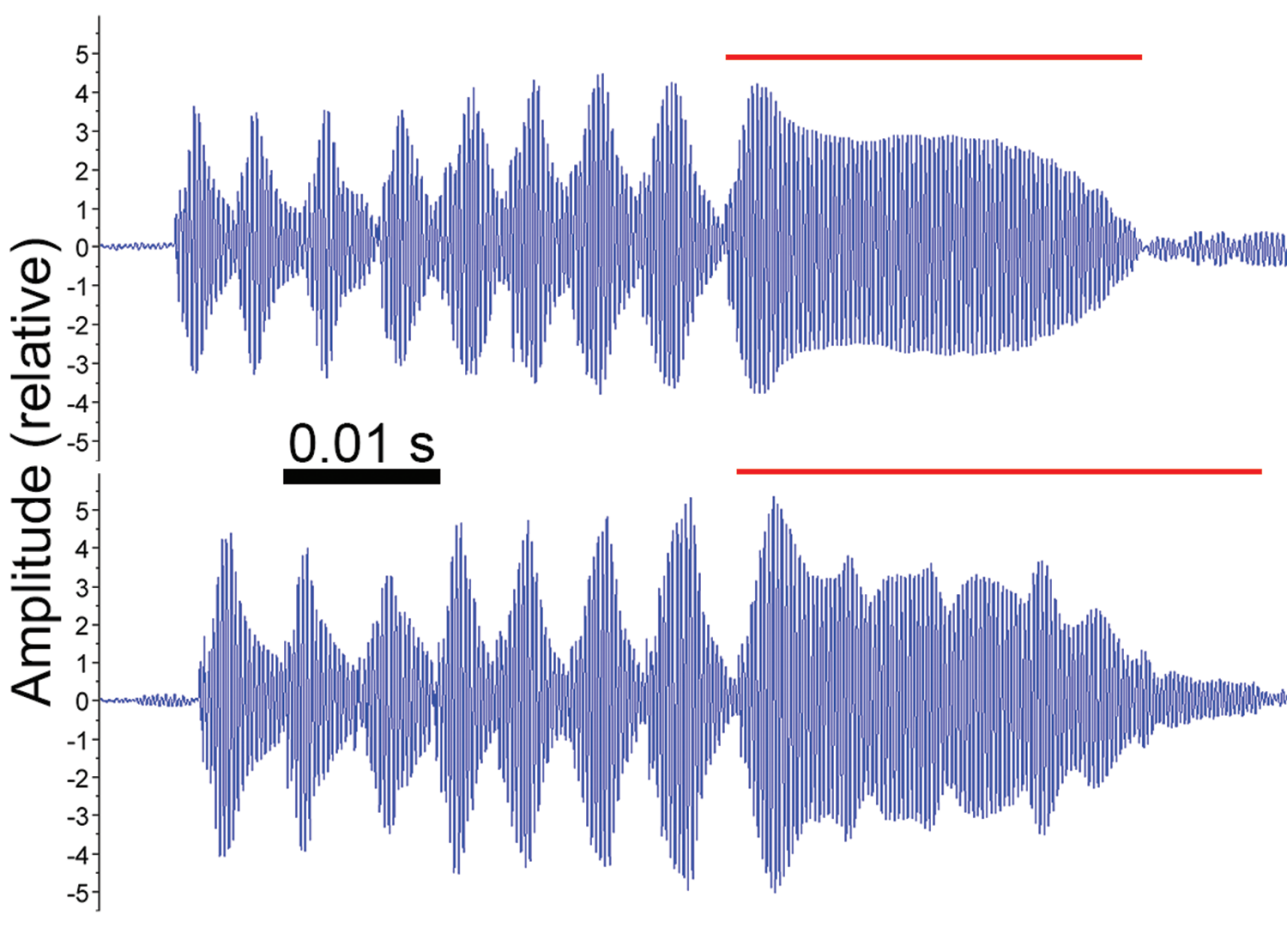
Calls of *Boanalatistriata* from its type locality. Two examples of call A, emitted by the individual ZUFRJ 15077 (snout-vent length = 40.0 mm), showing the last pulses fused in a pulsatile (below the red lines).

Call A was composed of a single, short, pulsed note, with up to seven visible harmonics and duration from 0.042 to 0.093 s (mean = 0.073 s, SD = 0,012, n = 97 calls; Fig. 1C, D). On an FFT size of 256 (270 Hz 3 dB filter bandwidth), sidebands are visible between the harmonics, caused by the pulse rate of the call. The dominant frequency was equal to the fundamental frequency, varying from 3375.0 to 3937.5 Hz (mean = 3626.3 Hz, SD = 168.2, Mo = 3562.5 Hz, n = 97 calls). Each call was composed of 11 to 21 pulses (mean = 15.5 pulses, SD = 2.4, Mo = 15 pulses, n = 68 calls) separated by intervals that were not fully silent, resulting in pulse duration equal to pulse period, which varied from 0.001 to 0.010 s (mean = 0.005 s, SD = 0.001, n = 1049 pulses). Each pulse had a rise time from 0.001 to 0.005 s (mean = 0.002 s, SD = 0.001, n = 1049 pulses), corresponding to 13.8–89.2% of the pulse duration (mean = 36.2%, SD = 8.7, n = 1049 pulses). A slightly ascending-descending frequency modulation is visible from the first to the last pulses, with the first pulse dominant frequency from 3000.0 to 3937.5 Hz (mean = 3547.3 Hz, SD = 190.0, Mo = 3562.5 Hz, n = 68 pulses), central pulse dominant frequency from 3375.0 to 4031.2 Hz (mean = 3745.9 Hz, SD = 143.5, Mo = 3843.8 Hz, n = 68 pulses), and last pulse dominant frequency from 3000.0 to 3843.8 Hz (mean = 3438.4 Hz, SD = 218.1, Mo = 3375.0 Hz, n = 68 pulses). In one individual (ZUFRJ 15077), all calls of this type presented some pulses fused in a pulsatile (Fig. 2), which made it impossible to count pulse number and to measure pulse parameters properly. Table 1 shows the parameters for each recorded male.

**Table 1.** Call parameters for the call A of each recorded individual of *Boanalatistriata*. Values are given as range, mean, standard deviation (SD), mode (Mo; when applicable) and sample number (n). Abbreviations: SVL, snout-vent length; CV_w_, within-individual coefficient of variation; CV_b_, between-individual coefficient of variation; CD, call duration; DF, dominant frequency; FF, fundamental frequency; PD, pulse duration; PP, pulse period; RT, pulse rise time; RTR, proportion of pulse rise time in relation to pulse duration; FPDF, first pulse dominant frequency; CPDF, central pulse dominant frequency; LPDF, last pulse dominant frequency; PN, pulse number. * All instances of call A of this individual presented some pulses fused in a pulsatile, which made it impossible to measure pulse parameters properly.

Parameters (Call A)	Individuals				Mean C_V_w	C_V_b
	ZUFRJ 15073 SVL = 35.4 mm	ZUFRJ 15074 SVL = 39.2 mm	ZUFRJ 15076 SVL = 39.3 mm	ZUFRJ 15077 SVL = 40.0		
CD (s)	0.066–0.089	0.067–0.078	0.077–0.094	0.042–0.078	7.7%	16.2%
	mean = 0.080	mean = 0.072	mean = 0.084	mean = 0.059		
	SD = 0.006; n = 31	SD = 0.003; n = 15	SD = 0.004; n = 22	SD = 0.008; n = 29		
	CV_w_ = 7.1%	CV_w_ = 4.7%	CV_w_ = 5.2%	CV_w_ = 13.6%		
PD (s) (= PP)	0.003–0.011	0.001–0.011	0.001–0.008	*	20.2%	24.0%
	mean = 0.006	mean = 0.005	mean = 0.005			
	SD = 0.001; n = 434	SD = 0.001; n = 215	SD = 0.001; n = 406			
	CV_w_ = 18.5%	CV_w_ = 22.3%	CV_w_ = 19.9%			
PN	11–16	11–17	17–21	*	8.4%	15.5%
	mean = 13.9	mean = 14.3	mean = 18.5			
	SD = 1.1	SD = 1.5	SD = 1.1			
	Mo = 15; n = 31	Mo = 15; n = 15	Mo = 18; n = 22			
	CV_w_ = 8.1%	CV_w_ = 10.8%	CV_w_ = 6.2%			
RT (s)	0.001–0.004	0.001–0.005	0.001–0.003	*	25.7%	25.0%
	mean = 0.002	mean = 0.002	mean = 0.002			
	SD = 0.001; n = 434	SD = 0.000; n = 215	SD = 0.000; n = 406			
	CV_w_ = 23.9%	CV_w_ = 24.4%	CV_w_ = 28.8%			
RTR (%)	13.8–89.2	18.2–64.3	15.4–63.6	*	21.0%	24.1%
	mean = 37.5	mean = 37.4	mean = 34.3			
	SD = 11.5; n = 434	SD = 5.6; n = 215	SD = 5.9; n = 406			
	CV_w_ = 30.8%	CV_w_ = 15.1%	CV_w_ = 17.1%			
DF (Hz) (= FF)	3656.2–3937.5	3656.2–3843.8	3375.0–3656.2	3375.0–3562.5	2.1%	4.6%
	mean = 3807.5	mean = 3712.5	mean = 3536.9	mean = 3455.8		
	SD = 92.8	SD = 69.1	SD = 71.9	SD = 69.6		
	Mo = 3750.0; n = 31	Mo = 3656.2; n = 15	Mo = 3562.5; n = 22	Mo = 3468.8; n = 29		
	CV_w_ = 2.5%	CV_w_ = 1.9%	CV_w_ = 2.0%	CV_w_ = 2.0%		
FPDF (Hz)	3375.0–3937.5	3562.5–3750.0	3000.0–3468.8	*	2.9%	5.4%
	mean = 3668.3	mean = 3618.7	mean = 3328.1			
	SD = 132.0	SD = 69.1	SD = 107.3			
	Mo = 3656.2; n = 31	Mo = 3562.5; n = 15	Mo = 3375.0; n = 22			
	CV_w_ = 3.6%	CV_w_ = 1.9%	CV_w_ = 3.2%			
CPDF (Hz)	3656.2–4031.2	3656.2–3843.8	3375.0–3750.0	*	2.1%	3.8%
	mean = 3861.9	mean = 3737.5	mean = 3588.1			
	SD = 85.3	SD = 69.7	SD = 77.5			
	Mo = 3843.8; n = 31	Mo = 3750.0; n = 15	Mo = 3562.5; n = 22			
	CV_w_ = 2.2%	CV_w_ = 1.9%	CV_w_ =2.2%			
LPDF (Hz)	3468.8–3843.8	3375.0–3468.8	3000.0–3468.8	*	2.5%	6.3%
	mean = 3634.9	mean = 3393.8	mean = 3191.8			
	SD = 95.9	SD = 38.8	SD = 124.4			
	Mo = 3656.2; n = 31	Mo = 3375.0; n = 15	Mo = 3187.5; n = 22			
	CV_w_ = 2.6%	CV_w_ = 1.1%	CV_w_ = 3.9%			

Call B was composed of a single, long, pulsed note, with up to seven visible harmonics and call duration from 0.711 to 1.610 s (mean = 1.114 s, SD = 0.279, n = 37 calls; Fig. 1E, F). The dominant frequency was equal to the fundamental frequency, varying from 3281.2 to 3750.0 Hz (mean = 3435.8 Hz, SD = 140.1, Mo = 3468.8 Hz, n = 37 calls). Each call was composed of 11 to 29 pulses (mean = 17.8 pulses, SD = 4.5, Mo = 17, n = 37 calls) separated by a fully silent interval. Pulse duration varied from 0.003 to 0.013 s (mean = 0.008 s, SD = 0.002, n = 682 pulses) and pulse period varied from 0.009 to 0.196 s (mean = 0.065 s, SD = 0.027, n = 645 periods). Each pulse had a rise time from 0.001 to 0.004 s (mean = 0.002 s, SD = 0.000, n = 682 pulses), corresponding to 14.2–51.6% of the pulse duration (mean = 26.9, SD = 5.7, n = 682 pulses). A slightly ascending-descending frequency modulation is visible from the first to the last pulses, with first pulse dominant frequency from 3000.0 to 3562.5 Hz (mean = 3310.9, SD = 138.0, Mo = 3375.0 Hz, n = 38 pulses), central pulse dominant frequency from 3187.5 to 3750.0 Hz (mean = 3473.6 Hz, SD = 178.6, Mo = 3468.8 Hz, n = 38 pulses), and last pulse dominant frequency from 2625.0 to 3656.2 Hz (mean = 3399.7 Hz, SD = 195.8, Mo = 3375.0 Hz, n = 38 pulses). Table 2 shows the parameters for each recorded male.

**Table 2.** Call parameters for the call B of each recorded individual of *Boanalatistriata*. Values are given as range, mean, standard deviation (SD), mode (Mo; when applicable) and sample number (n). Abbreviations: SVL, snout-vent length; CV_w_, within-individual coefficient of variation; CV_b_, between-individual coefficient of variation; CD, call duration; DF, dominant frequency; FF, fundamental frequency; PD, pulse duration; PP, pulse period; RT, pulse rise time; RTR, proportion of pulse rise time in relation to pulse duration; FPDF, first pulse dominant frequency; CPDF, central pulse dominant frequency; LPDF, last pulse dominant frequency; PN, pulse number. Mode followed by an “-“ indicates that no value was more frequent than the others.

Parameters (Call B)	Individuals				Mean CV_w_	CV_b_
	ZUFRJ 15073 SVL = 35.4 mm	ZUFRJ 15074 SVL = 39.2 mm	ZUFRJ 15076 SVL = 39.3 mm	ZUFRJ 15077 SVL = 40.0		
CD (s)	1.030–1.234	1.301–1.476	1.564–1.610	0.711–0.894	4.5%	25.1%
	mean = 1.130	mean = 1.384	mean = 1.577	mean = 0.814		
	SD = 0.073; n = 12	SD = 0.550; n = 8	SD = 0.022; n = 4	SD = 0.048; n = 14		
	CV_w_ = 6.5%	CV_w_ = 4.0%	CV_w_ = 1.4%	CV_w_ = 5.9%		
PD (s)	0.003–0.012	0.001–0.010	0.003–0.010	0.004–0.013	16.9%	21.2%
	mean = 0.007	mean = 0.008	mean = 0.008	mean = 0.009		
	SD = 0.002; n = 214	SD = 0.001; n = 170	SD = 0.001; n = 106	SD = 0.001; n = 192		
	CV_w_ = 26.4%	CV_w_ = 14.6%	CV_w_ = 13.0%	CV_w_ = 13.4%		
PP (s)	0.009–0.152	0.024–0.166	0.024–0.121	0.026–0.196	38.5%	41.4%
	mean = 0.066	mean = 0.068	mean = 0.062	mean = 0.063		
	SD = 0.027; n = 203	SD = 0.021; n = 162	SD = 0.017; n = 102	SD = 0.035; n = 178		
	CV_w_ = 40.7%	CV_w_ = 31.1%	CV_w_ = 27.2%	CV_w_ = 54.9%		
PN	15–24	20–23	24–29	11–16	9.1%	25.3%
	mean = 17.4	mean = 21.3	mean = 26.5	mean = 13.7		
	SD = 2.3	SD = 1.0	SD = 2.4	SD = 1.2		
	Mo = 17; n = 12	Mo = 21; n = 8	Mo = -; n = 4	Mo = 14; n = 14		
	CV_w_ = 13.5%	CV_w_ = 4.9%	CV_w_ = 9.0%	CV_w_ = 8.8%		
RT (s)	0.001–0.003	0.001–0.002	0.001–0.002	0.001–0.004	16.2%	20.0%
	mean = 0.002	mean = 0.002	mean = 0.002	mean = 0.002		
	SD = 0.001; n = 214	SD = 0.000; n = 170	SD = 0.000; n = 106	SD = 0.000; n = 192		
	CV_w_ = 24.2%	CV_w_ = 12.7%	CV_w_ = 14.7%	CV_w_ = 13.1%		
RTR (%)	18.8–51.6	14.8–50.0	16.3–50.0	14.2–44.2	16.5%	21.2%
	mean = 31.9	mean = 24.7	mean = 25.5	mean = 24.0		
	SD = 5.6; n = 214	SD = 4.1; n = 170	SD = 4.0; n = 106	SD = 3.9; n = 192		
	CV_w_ = 17.7%	CV_w_ = 16.6%	CV_w_ = 15.8%	CV_w_ = 16.1%		
DF (Hz) (= FF)	3281.2–3750.0	3375.0–3468.8	3468.8	3281.2–3375.0	1.5%	4.1%
	mean = 3579.5	mean = 3457.1	min = max = mean = Mo	mean = 3301.3		
	SD = 137.9	SD = 33.2	SD = 0.0	SD = 39.9		
	Mo = 3656.2; n = 12	Mo = 3468.8; n = 8	n = 4	Mo = 3281.2; n = 14		
	CV_w_ = 3.9%	CV_w_ = 1.0%	CV_w_ = 0.0%	CV_w_ = 1.2%		
FPDF (Hz)	3187.5–3562.5	3375.0–3468.8	3093.8–3187.5	3000.0–3375.0	2.4%	4.2%
	mean = 3414.1	mean = 3386.7	mean = 3140.7	mean = 3227.7		
	SD = 123.0	SD = 33.2	SD = 54.1	SD = 102.1		
	Mo = 3562.5; n = 12	Mo = 3375.0; n = 8	Mo = 3187.5; n = 4	Mo = 3281.2; n = 14		
	CV_w_ = 3.6%	CV_w_ = 1.0%	CV_w_ = 1.7%	CV_w_ = 3.2%		
CPDF (Hz)	3562.5–3750.0	3468.8	3468.8	3187.5–3375.0	0.9%	5.1%
	mean = 3703.1	min = max = mean = Mo	min = max = mean = Mo	mean = 3293.7		
	SD = 63.2	SD = 0.0	SD = 0.0	SD = 60.00		
	Mo = 3750.0; n = 12	n = 8	n = 4	Mo = 3281.2; n = 14		
	CV_w_ = 1.7%	CV_w_ = 0.0%	CV_w_ = 0.0%	CV_w_ = 1.8%		
LPDF (Hz)	2625.0–3656.2	3281.2–3468.8	2906.2–3468.8	3281.2–3468.8	4.7%	5.8%
	mean = 3507.8	mean = 3386.7	mean = 3210.9	mean = 3368.3		
	SD = 283.9	SD = 60.1	SD = 234.4	SD = 57.8		
	Mo = 3562.5; n = 12	Mo = 3375.0; n = 8	Mo = -; n = 4	Mo = 3375.0; n = 14		
	CV_w_ = 8.1%	CV_w_ = 1.8%	CV_w_ = 7.3%	CV_w_ = 1.7%		

In general, spectral parameters were static, while temporal parameters were dynamic. The most static parameter was the dominant frequency (mean CV_w_ and CV_b_ was 2.1% and 4.6% in call A and 1.5% and 4.1% in call B, respectively), while pulse period of call B was the most dynamic (CV_w_ and CV_b_ was 38.5% and 41.4%, respectively). Some parameters, including first pulse dominant frequency and last pulse dominant frequency in call A and central pulse dominant frequency and last pulse dominant frequency in call B, were intermediate in CV_b_, but static in CV_w_. All CV_w_ and CV_b_ values are shown in Tables 1 and 2.

A fight event between two males of *B.latistriata* was witnessed during the recordings. A male (M1) was calling from the marginal vegetation, perched at approximately 20 cm from the water surface. A second male (M2) was calling from a floating shrub at approximately 40 cm from M1. Without any previous alteration on vocalization, M1 jumped over M2 and started the fight, both grasping and kicking. This first round lasted less than a second and resulted in M2 moving approximately 20 cm away. After a few seconds, M1 pursued M2, starting a second round of fighting, which lasted about the same time as the first one. After this, M2 swam to the other side of the pond, where it started calling a few minutes later. M1 returned to its original calling site and started calling again immediately. M1 was then recorded and collected, and is one of the individuals included in this study (ZUFRJ 15076). Individual M2 was not recorded nor collected.

## Discussion

In this study, we describe two calls of *B.latistriata* and the coefficient of variation of its parameters. A fight event between two males is also reported. Although males of the species of the *B.polytaenia* clade frequently present dorsum marks that suggest fighting (C. Luna-Dias pers. comm.), this is the first published record of a fight for the clade. Although both intraspecific (Giasson and Haddad 2006, Toledo et al. 2007, Nali and Prado 2014) and interspecific (Reichert and Gerhardt 2014, Guerra et al. 2018b) fighting events are broadly reported for anurans, the event reported here was short when compared to similar fights in other species (for example, Nali and Prado 2014, Fernandes et al. 2018).

The calls of *B.latistriata* are similar to those of other species of the *B.polytaenia* clade. By presenting a shorter, single-note call with lower pulse period (call A), and a longer call with higher pulse period (call B), it resembles the calls from *B.bandeirantes*, *B.beckeri*, *B.polytaenia*, and B.aff.beckeri (Acioli and Toledo 2008, Pinheiro et al. 2012). In these characteristics, it differs from the described calls of *B.botumirim* (call B absent; Caramaschi et al. 2009), *B.cipoensis* (call A composed of 1–3 notes; Batista et al. 2015), *B.goiana* (call A composed of two notes; Menin et al. 2004), *B.jaguariaivensis* (call A composed of 1–4 notes; Guerra et al. 2017), *B.leptolineata* (call B absent; Kwet 2001), *B.phaeopleura* (call A composed of 2–5 notes; Pinheiro et al. 2012), and *B.stenocephala* (call A composed of 2–4 notes; Martins et al. 2016). The call A of *B.latistriata* also differs from some of these species in the pulse number: 11–21 in *B.latistriata* (present study), 3–5 in *B.botumirim* (Caramaschi et al. 2009), 1–5 in *B.jaguariaivensis* (Guerra et al. 2017), and 2–5 in *B.stenocephala* (Martins et al. 2016).

The calls of *B.latistriata* are distinguished from those of the four species whose calls are most similar to it by the lower dominant frequency of both calls A and B: 3375.0–3937.5 and 3281.2–3750.0 Hz, respectively, in *B.latistriata* (present study); 5340.2–5857.0 and 5340.2–5512.5 Hz, respectively, in *B.bandeirantes* (Pinheiro et al. 2012); 3938.0–5063.0 and 3938.0–4875.0 Hz, respectively, in *B.beckeri* (Martins et al. 2016); 6890.0–7320.0 and 6460.0–7320.0 Hz, respectively, in B.aff.beckeri (Acioli and Toledo 2008). These species are also morphologically similar (Caramaschi and Cruz 2013), and hence, the use of calls for identifying species and clarifying phylogenetic relationships must be encouraged. Additionally, of all species of the *B.polytaenia* clade, frequency modulation was previously reported only for *B.goiana* (Menin et al. 2004). However, in this species the frequency ascends from the first to the last pulses, while in *B.latistriata* the frequency ascends from the first to the central pulses, thereafter decreasing to the last pulses.

Values of CV are linked to issues like recognition (at the species, population, and individual levels) and female preferences, and comparing those coefficients can be taxonomically informative (Köhler et al. 2017). Gerhardt (1991) stated that static CV_w_ parameters may be linked with recognition. All spectral parameters, as well as call B duration, were static on a within-individual basis. Furthermore, CV_b_ values much greater than CV_w_ indicates parameters useful for individual recognition (Gambale et al. 2014, Forti et al. 2014, Köhler et al. 2017). Call B duration had CV_b_ 5.6 times greater than CV_w_ and is the parameter that best fits this purpose. The finding that spectral parameters were static and temporal parameters were dynamic is congruent with Köhler et al. (2017) and with the results reported by Guerra et al. (2017) for *B.jaguariaivensis*. However, call B duration being static at the within-individual level is a novelty for the *B.polytaenia* clade. As social context can influence call duration in some anurans (Gambale and Bastos 2014, Gambale et al. 2014), social interaction experiments involving *B.latistriata* will be useful to understand this variation.

Despite the similarities between the calls of the species of the *B.polytaenia* clade, different terminology used in call descriptions can lead to difficulties in comparing those descriptions if the terminology is not well explained. For example, structures of the call B herein defined as “pulses” were called as “notes” by Acioli and Toledo (2008) and by Pinheiro et al. (2012). In these cases, the terminology is well stated, and can be comprehended without ambiguity. However, the lack of clear definitions for the calls of *B.cipoensis* and *B.leptolineata* (Kwet 2001; Batista et al. 2015) resulted in few possible comparisons. The redescription of the calls of these species, as well as the description of the call of *B.buriti*, will make possible a full comparison of the calls in the *B.polytaenia* clade, serving as a powerful tool for the taxonomy of this clade.
